# Early mobilization after uncomplicated medial subtalar dislocation provides successful functional results

**DOI:** 10.1007/s10195-011-0126-2

**Published:** 2011-02-10

**Authors:** Nikolaos G. Lasanianos, Dimitrios N. Lyras, George Mouzopoulos, Nikolaos Tsutseos, Christos Garnavos

**Affiliations:** 1 Athens General Infirmary “Evangelismos”, 45-47 Ypsilantou str., 10676 Athens, Greece; 2 Athens General Hospital “Amalia Fleming”, Athens, Greece

**Keywords:** Subtalar joint, Dislocations, Ankle, Foot, Rehabilitation

## Abstract

**Background:**

Subtalar dislocation is a rare injury, with the medial type occurring in the majority of cases. The period of postreduction immobilization is a matter of controversy. Most studies set the period of immobilization between 4 and 8 weeks. The hypothesis in this study is that a period of 2–3 weeks of immobilization in a cast, followed by early mobilization, could provide better functional results than longer periods of immobilization.

**Materials and methods:**

During a period of 4 years, eight patients (six men, two women) with mean age of 37.2 years and uncomplicated medial subtalar dislocation were treated in our institution. Immediate reduction under sedation and cast immobilization was provided in all cases. Our rehabilitation protocol consisted of two completed weeks of immobilization and thereafter ankle range-of-motion exercises and partial weight-bearing mobilization. Patients were followed up for a mean period of 3 years. Clinical results were evaluated using the AOFAS Ankle–Hindfoot scale.

**Results:**

All patients achieved almost normal ankle range of motion and good clinical outcome (mean AOFAS score 92.25). No radiographic evidence of arthritis or avascular necrosis of the talus was detected. Two patients complained of mild pain of the hindfoot. All patients returned to daily routine activities in about 2 months from injury.

**Conclusions:**

Immediate reduction and early mobilization could be key factors for uneventful recovery of uncomplicated medial subtalar dislocation. Multicenter clinical trials are needed for further validation of our initial results.

**Level of evidence:**

III, prospective clinical series study.

## Introduction

Subtalar dislocation (StD) is an uncommon type of injury that involves concomitant loss of normal anatomical relations between talus, navicular, and calcaneus, while the tibiotalar and calcaneocuboid joints remain congruent [1, 2]. The mechanism of StD is trauma to a plantar-flexed foot either in inversion, resulting in medial subtalar joint dislocation (85%), or in eversion, resulting in lateral dislocation (15%). Anterior and posterior dislocations have also been described but are exceedingly rare [3, 4]. Immediate reduction is of paramount importance and is usually provided under sedation. It is followed by a period of immobilization necessary for the healing of the soft tissues. The majority of studies specify this period of immobilization between 4 and 8 weeks [5–10]. However, it has also been stated that subtalar joint stiffness in uncomplicated StD can be minimized by avoiding immobilization longer than 4 weeks [7–10]. In general, the duration of postreduction immobilization, which correlates with the amount of stiffness and remaining functionality, is a controversial subject. We conducted this study to reassess the optimal duration of the immobilization period, after uncomplicated medial StD, able to provide both subtalar joint stability and avoidance of stiffness. Our working hypothesis was that a period of 2–3 weeks of immobilization in a cast, followed by range-of-motion (ROM) exercises and partial weight bearing (PWB), could provide better functional results than those achieved by longer periods of immobilization. To increase the validity of our outcome we chose to use a prespecified treatment and rehabilitation protocol, creating a prospective study. To the best of our knowledge, previous researchers have only retrospectively examined this type of injury.

## Materials and methods

**Fig. 1:**
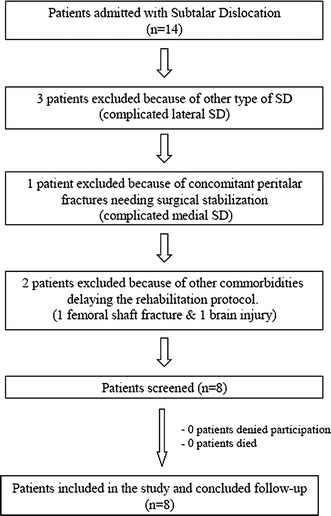
Flow diagram of patients enrolled in the study

This prospective study concerned a period from June 2004 through March 2008. The research was approved by the local Ethics Committee, and all patients signed informed consent. The study was performed in accordance with the ethical standards of the 1964 Declaration of Helsinki as revised in 2000. Men or women of any age admitted for StD were considered for inclusion in the study. The inclusion criteria included: (1) medial subtalar dislocation, and (2) open or closed injuries. Patients were excluded if they had: (1) peritalar fracture accompanying the dislocation, (2) subtalar dislocation of other type (lateral, anterior, posterior), or (3) other comorbidities that would influence or delay the rehabilitation protocol (e.g., brain damage, bilateral lower limbs injuries). Of the 14 patients examined in the Accidents and Emergency (A & E) department, 8 fulfilled the inclusion criteria and were enrolled in the study (Fig. 1). Of those eight patients, six were male and two were female (male-to-female ratio of 3:1). The mean age of the patients was 37.25 years (range 25–54 years). Five of the patients (62.5%) had undergone a motor vehicle accident (MVA), two patients (20%) had sustained a fall from height, and another one (12.5%) an inversion injury when his foot was trapped in a gap. All injuries were neurovascular intact, including seven closed and one open medial subtalar dislocation.

Treatment was provided by immediate closed reduction in the A & E department under sedation. Axial traction on the foot and heel in the line of deformity was combined with countertraction with the knee in flexion to relax the gastrocnemius muscle. Abduction of the foot and dorsiflexion of the ankle followed. After reduction, the ankle and foot were immobilized in a below-the-knee backslab followed by administration of analgesia and foot elevation. The backslab was transformed to a below-the-knee jigsaw cast 3–4 days post reduction as long as the swelling had subsided and non-weight-bearing (NWB) mobilization was allowed. Active ankle and foot ROM exercises were initiated by the beginning of the third week from reduction. Partial weight-bearing began after the third week, progressing to full weight-bearing (FWB) mobilization by the fifth week. Weight-bearing mobilization was assisted by the use of a below-the-knee functional brace that allowed plantarflexion and dorsiflexion but restricted inversion and eversion movements. Muscle-strengthening physiotherapy was implemented with the beginning of ROM exercises.

The time intervals between injury, reduction, and initiation of the several mobilization stages were recorded. All patients followed the same early mobilization protocol. Clinical results were evaluated using the American Orthopaedic Foot and Ankle Society (AOFAS) Ankle–Hindfoot scale [11], which assigns 50 points to function, 40 to pain, and 10 to alignment of the foot. Moreover, a relative ankle ROM score was created for every patient to assess the post injury and immobilization remaining stiffness. This score consisted of a percentage resulting from the ratio of the ankle ROM on the injured leg in comparison with the contralateral healthy limb. The clinical measurements were accomplished by use of a goniometer. Since we were unable to obtain a perfectly accurate measurement, we preferred to create approximate ratios using steps of 5% (Table 1).

**Table 1:** Demographics and analytical results of the eight cases presented

Case no.	Age/gender	Mechanism of injury	Open/closed	Time from injury to reduction (min)	Time from injury to ROM exercises (days)	Time from injury to PWB mobilization (days)	Time from injury to FWB mobilization (days)	AOFAS score (function)	AOFAS score (pain)	AOFAS score (alignment)	AOFAS score (total)	Relative ankle ROM*	Follow-up (months)
1	M/25	MVA	Closed	90	15	23	35	44/50	40/40	8/10	92	95%	47
2	M/38	Inversion injury	Closed	120	16	21	34	44/50	40/40	10/10	94	90%	49
3	M/42	Fall from height	Closed	150	18	22	35	41/50	40/40	8/10	89	90%	36
4	F/33	MVA	Open	120	17	23	37	44/50	40/40	10/10	94	100%	37
5	M/54	MVA	Closed	150	18	24	38	44/50	30/40	10/10	84	90%	35
6	M/49	MVA	Closed	90	15	22	36	47/50	40/40	10/10	97	95%	34
7	F/28	Fall from height	Closed	120	16	24	36	44/50	40/40	10/10	94	95%	26
8	M/29	MVA	Closed	180	18	26	42	44/50	30/40	8/10	82	85%	24
Mean SD	37.25 ± 10.44			127.5 ± 31.05	16.625 ± 1.3	23.125 ± 1.55	36.625 ± 2.5	44 ± 1.6	37.5 ± 4.62	9.25 ± 1.03	90.75 ± 5.31	92.5% ± 4.62%	36 ± 8.78

* Percentage of ROM of the injured ankle in relation to the contralateral healthy ankle

## Results

**Fig. 2:**
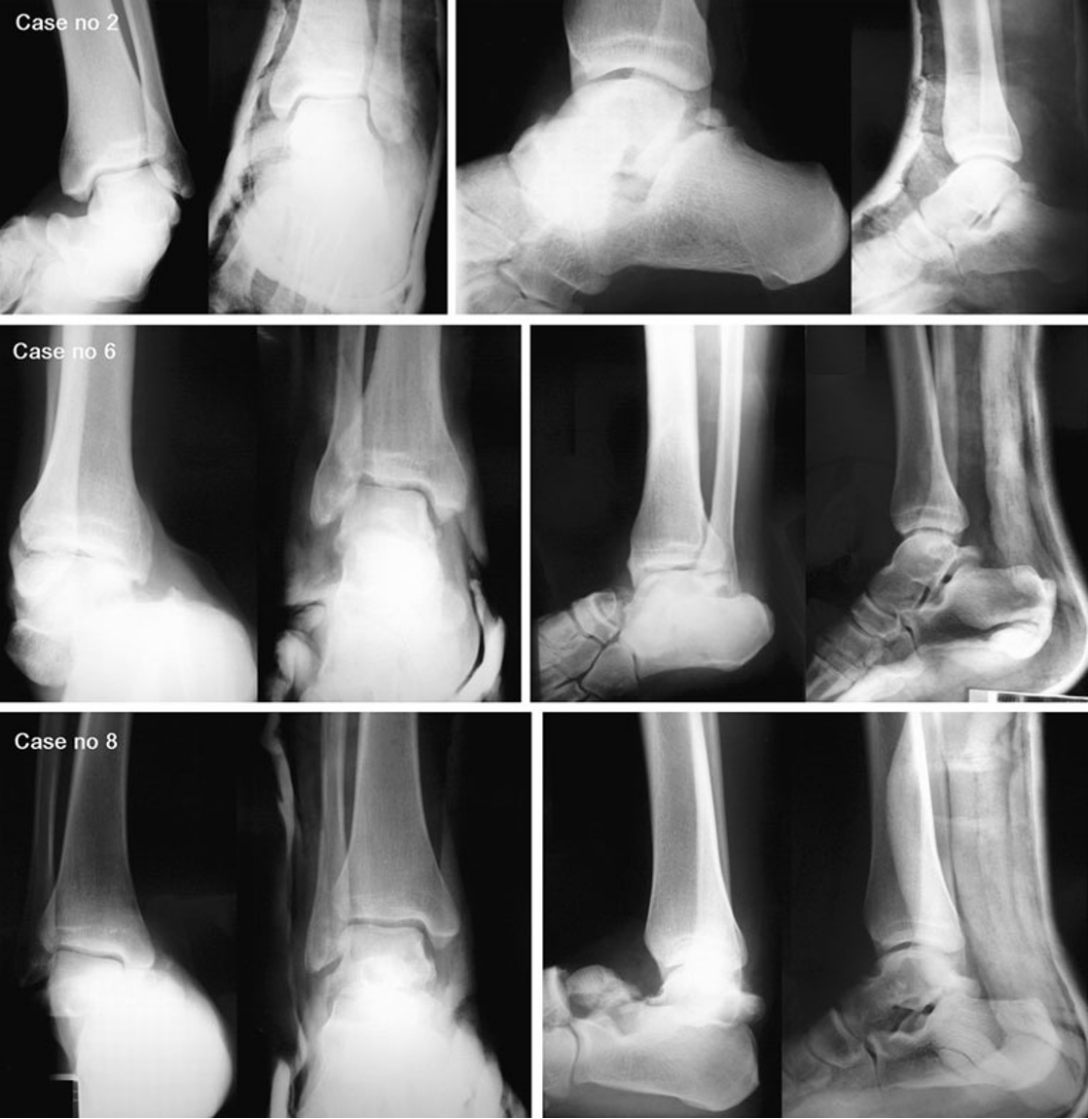
Pre- and postreduction anteroposterior and lateral radiographs of cases 2, 6, and 8

**Fig. 3:**
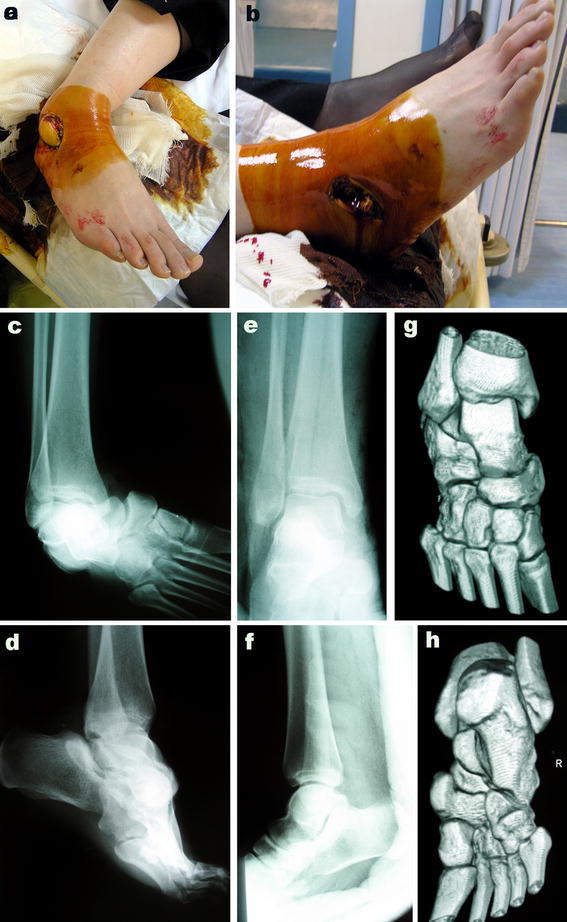
**a**, **b** Pre- and postreduction clinical views of the open medial subtalar dislocation of case 4. **c**–**f** Pre- and postreduction anteroposterior and lateral radiographs of the open medial subtalar dislocation of case 4. **g**, **h** Postreduction computed tomography with three-dimensional (3D) reconstruction views for the detection of any occult fracture

Successful closed immediate reduction under sedation was achieved for all cases (Figs. 2, 3). The mean time between injury and reduction was recorded to be 127.5 (range 90–180) min. In all cases the hindfoot was found to be well aligned with no signs of secondary instability under stress and a below-the-knee backslab was applied. No neurovascular damage was recorded pre or post reduction. For the case of the open dislocation, adequate washout was performed prior to reduction. The wound was left open under chemoprophylaxis and was secondary closed 3 days later during the jigsaw cast application. There was no need for a skin graft, since the wound was successfully sutured after debridement. Wound check was accomplished by periodical removal of the jigsaw cast.

**Fig. 4:**
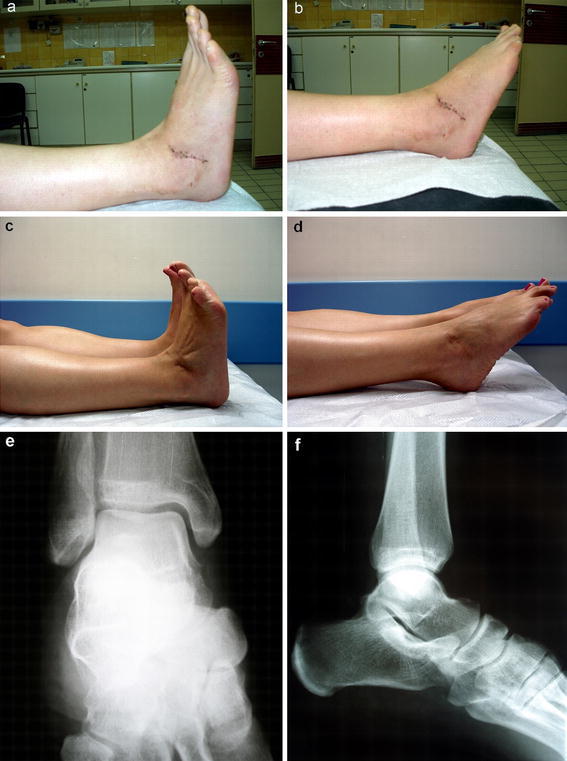
**a**, **b** Range of motion of the ankle joint 2 months post injury. **c**, **d** Range of motion of the ankle joint 3 years post injury. **e**, **f** Radiographic control 3 years post injury without evidence of arthritis or avascular necrosis

Follow-up assessment was conducted by clinical and radiographic examination every 2 months for the first 6 months post patient discharge. Thereafter, follow-up continued with yearly routine checks for a mean period of 36 months (range 24–49 months). The mean percentage of ankle ROM between the injured and the healthy lower limb (Fig. 4 a–d) was 92.5% (range 85–100%), which was considered as very satisfactory by both physicians and patients (Table 1). No radiographic evidence of arthritis or avascular necrosis of the talus was detected in any patient until the final follow-up appointment (Fig. 4e, f). The mean AOFAS score was 90.75 points (range 82–97) (Table 1). Two out of eight patients complained of transient mild pain which did not restrict them from their daily activities. One female patient correlated this pain with protracted walking on flat shoes, whereas heeled shoes did not seem to annoy her. None of the patients was keen on sports, preventing evaluation of ankle and foot functionality under conditions of repetitive stress. All patients returned to their previous professional occupation and were happy with the outcome.

## Discussion

Management of StD requires immediate reduction under sedation to avoid soft-tissue and vascular complications [1]. Closed reduction is usually successful [1, 12, 13]. In nonreducible cases, however, multiple reduction attempts using force should not be undertaken, and open reduction should be performed without delay [6]. Frequently, associated lesions occur in the ankle and foot such as osteochondral fractures of the dislocated articular surfaces and fractures of neighboring bones (malleoli, the base of the fifth metatarsal, the cuboid, and the navicular tuberosities) [14]. If such concomitant injuries are suspected from routine radiological images, further investigation should be undertaken with computed tomography (CT) scan.

We believe that the prognosis of an isolated medial subtalar dislocation basically relies on three parameters: (1) immediate reduction (the necessity of which has already been underlined), (2) the amount of energy absorbed by the soft tissues at the moment of the violent impact, and (3) the period of postreduction immobilization.

The need for immediate reduction has already been discussed earlier. The mechanism of injury is an important factor in predicting long-term results. The results are worse after more violent mechanisms [14]. Simple inversion rarely produces dislocation with long-term morbidity, while more violent injuries, e.g., those incurred in motor vehicle accidents or after a fall from height, are more likely associated with persistent symptoms [14]. Concerning the immobilization period, previous studies have set the length of non-weight-bearing plaster use to 5 or 6 weeks [5, 6]. According to other researchers [7–10], subtalar joint stiffness in uncomplicated StD can be minimized by avoiding immobilization longer than 4 weeks, or 6 weeks in case of StD associated with fractures. In general, the duration of postreduction immobilization is a controversial subject. Christiensen et al. [15] immobilized their patients in a long cast for 8 weeks after reduction of the dislocation. Twenty-one of their 30 patients faced pain when walking. Buckingham [16] reported on five patients, of whom four had decreased ROM following immobilization for 6 weeks. In other previous large series [5] of uncomplicated medial StD that incorporated immobilization for 5 weeks, subtalar joint ROM was decreased by 30–50% compared with the contralateral side and the tibiotalar ROM was moderately reduced. Moreover long immobilization has been correlated with high percentages of arthritis and decreased function rating between 50–80% [6, 15, 17]. McKeever [18], on the other hand, strongly encouraged measures to prevent fibrosis by early mobilization of the foot. He stated that, since the reduction of a subtalar dislocation is extremely stable, early mobilization is possible. He began ROM exercises, consisting of active assisted motion of the subtalar and midtarsal joints, after 3 weeks of immobilization. Of the eight patients he treated, five were immobilized for 3 weeks and had a normal range of subtalar motion and no complaint of pain. Forty-five years after McKeever’s study, our research confirms the advantages of early mobilization protocols for uncomplicated medial StD, using an even shorter period of immobilization than McKeever did.

The prospective nature of our study, which allowed for the use of a prespecified protocol, ensured better control of the patient cohort during the follow-up period and a more reliable survey of the protocol itself. The application of certain inclusion and exclusion criteria created a homogeneous group of patients (those who had sustained uncomplicated medial StD), excluding bias due to nonsimilar injury patterns. We preferred to focus solely on uncomplicated medial StD, since this is the most usual type of peritalar dislocation that a clinician may need to manage. Homogeneity was also ensured by the fact that all patients received the same reduction method, which consisted of manipulation under sedation. It should be noted that none of the patients needed to receive general anesthesia, as usually suggested in literature [1, 12, 13]. Sedation proved sufficient, allowing immediate reduction in the A & E department and saving valuable time that would be lost if the patient had to be transferred to theater and receive general anesthesia. Another strong point of our study was the existence of pre-assessed intervals for clinical and radiographic follow-up examination, which allowed for analogue timescale comparisons to be made. In contrast, the limited number of cases was a drawback of our research; this was expected, since subtalar dislocation is a rare injury. The small series of patients was the price to pay for the prospective character of the study. We believe, however, that it was worthwhile since the results, albeit from a small sample, were not controversial. Nonetheless, the study is still ongoing, thus further patients and results will be added in the future. Additional credibility could have been provided to the study if a control group of patients undergoing longer periods of immobilization had been included. The researchers were limited by two factors: the already mentioned small number of cases, and the ethical hesitation to provide patients with a type of treatment (long periods of immobilization) that we did not believe to be optimal.

Based on the aforementioned, it would be safe to say that our working hypothesis was confirmed: Early ankle ROM exercises and PWB mobilization after uncomplicated medial StD seem to provide better functional results than those achieved by longer periods of immobilization, as already mentioned in literature. As joint instability, following closed reduction of medial subtalar dislocation, is not a possible complication, protracted immobilization only adds to joint stiffness and minimizes ankle and foot functionality. In contrast, early active ROM exercises may help the ligaments and the tendons of the site to heal without compromising proprioception of the joint.

As basic practical recommendations extracted from this study, we could state the following: (a) immediate closed reduction should be applied in the case of any kind of StD, (b) sedation of the patient is usually sufficient for reduction of uncomplicated medial StD, (c) time-consuming general anesthesia should be used only in case of irreducible closed StD requiring open surgical reduction procedures, and (d) early mobilization protocols are indicated as beneficial for ankle and foot functionality after medial uncomplicated StD. Continuation of this study will add further credibility to its usefulness. Nonetheless, for definite results to be drawn, multicenter clinical trials will be required, and the creation of collaborative databanks of patients between multiple centers and countries may be necessary.

## Abbreviations

AOFAS: American Orthopaedic Foot and Ankle Society (AOFAS)
FWB: Full weight bearing
MVA: Motor vehicle accident
PWB: Partial weight bearing
ROM: Range of motion
SD: Standard deviation
StD: Subtalar dislocation
